# FLIP Measurements Predict Short‐Term Outcomes After Singular EsoFLIP Dilation in Patients With Achalasia

**DOI:** 10.1111/nmo.70500

**Published:** 2026-11-09

**Authors:** Johann Stuby, Valeria Schindler, Joel Schnellmann, Jessica Martinez, Amanda Bianca, Daniel Pohl

**Affiliations:** ^1^ Department of Gastroenterology and Hepatology Zurich University Hospital Zurich Switzerland; ^2^ Department of Internal Medicine Stadtspital Triemli Zurich Switzerland

**Keywords:** cross‐sectional area, distensibility index, dysphagia, endoscopic dilation, esophageal motility disorder

## Abstract

**Background:**

FLIP enables real‐time measurement of esophagogastric junction (EGJ) dimensions and pressures during dilation. While FLIP metrics predict outcomes after pneumatic dilation, POEM, and myotomy, their predictive value following EsoFLIP dilation remains unknown. This study identified FLIP measurements predicting short‐term outcomes after EsoFLIP dilation in patients with achalasia.

**Methods:**

Treatment‐naïve patients undergoing singular EsoFLIP dilation (2017–2025) were analyzed. EGJ diameter (EGJ‐Dia) and cross‐sectional area (EGJ‐CSA) were measured at 70 mL, distensibility index (EGJ‐DI) and balloon pressure (EGJ‐BP) at 60 mL, pre‐ and post‐dilation. Primary outcome was excellent short‐term symptomatic response at 4 weeks (Eckardt score ≤ 1).

**Results:**

Seventy‐nine patients (38% female, median age 52 years, achalasia type 1: 8%, type 2: 87%, type 3: 5%) were included. Symptomatic response was 81% (ES ≤ 3 or Δ ≥ 3) and 46% (ES ≤ 1); objective response 67%. Median ES decreased from 6 (IQR 4–7) to 2 (IQR 0–4). Median EGJ‐Dia increased from 10 mm to 17 mm, EGJ‐CSA from 72 mm^2^ to 220 mm^2^, and EGJ‐DI from 1.4 mm^2^/mmHg to 5.8 mm^2^/mmHg, while EGJ‐BP decreased from 31 mmHg to 24 mmHg (all *p* < 0.001). Among 51 patients with complete data, ΔEGJ‐Dia70 (AUROC 0.77, cut‐off ≥ 6.3 mm) and ΔEGJ‐CSA70 (AUROC 0.75, cut‐off ≥ 129.3 mm^2^) showed highest predictive performance. ΔEGJ‐DI60 showed moderate performance (AUROC 0.65), while ΔBP60 was non‐discriminative (AUROC 0.45).

**Conclusion:**

Delta values in FLIP metrics, particularly ΔEGJ‐Dia and ΔEGJ‐CSA, predicted excellent short‐term symptomatic response. These findings indicate that intra‐procedural FLIP panometry may help assess treatment adequacy during EsoFLIP dilation.

## Plain Language Summary

Achalasia is a swallowing disorder characterized by aperistalsis and importantly failure of LES relaxation. Treatment options focus on opening this tight junction that obstructs flow by different measures. A new dilation technique called FLIP uses a special balloon to treat and measure the junction's opening in real‐time during the procedure. This study examined 79 patients to determine which parameters best predict treatment success after EsoFLIP dilation. We found that the change in junction diameter (increase of at least 6.3 mm) and cross‐sectional area (increase of at least 129 mm^2^) during dilation were the best predictors of excellent symptom improvement at 4 weeks. These findings suggest that real‐time measurements during the procedure can help guide how much dilation is needed to optimize treatment success.

## Key Points

**What Was Known?:**

- EsoFLIP dilation is increasingly employed in the treatment of esophageal structural and motility disorders [1, 2].
- FLIP metrics (DI, Dia, CSA) may predict outcomes after POEM, LHM, and PD [3–8].
- Standardized FLIP protocol (Dallas Consensus: 50–60–70 mL) improves comparability [9].

**What This Study Adds?:**

- First predictive FLIP data after EsoFLIP dilation in achalasia.
- Delta flip metrics are predictors of short‐term outcomes: ΔEGJ‐DI60 3.5 mm^2^/mmHg (AUC 0.65), ΔEGJ‐Dia70 6.3 mm (AUC 0.77).
- ΔEGJ‐CSA70 as complementary predictor (cut‐off 129.3 mm^2^, AUC 0.75).

## Graphical abstract

Changes in EGJ diameter and EGJ cross‐sectional area during EsoFLIP dilation predict short‐term symptomatic response. Intra‐procedural FLIP may help assess treatment adequacy, but prospective validation is required before clinical application.

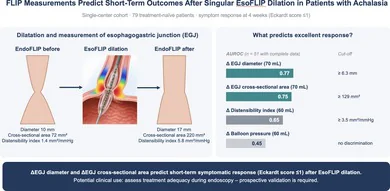

## 1 Introduction

Achalasia is a chronic esophageal motility disorder characterized by absent peristalsis and insufficient relaxation of the lower esophageal sphincter (LES), leading to progressive dysphagia, regurgitation, and impaired quality of life. Current treatment options aim to reduce LES pressure and include pneumatic dilation (PD), peroral endoscopic myotomy (POEM), and laparoscopic Heller myotomy (LHM), with comparable short‐ to medium‐term efficacy [1, 2, 3] in subtypes I and II.

PD has been the mainstay of endoscopic treatment for decades, using balloons of progressively larger diameter (typically 30 mm, possibly 35 mm and less commonly 40 mm). The need for repeat dilation with a step‐up in balloon size can only be determined at post‐procedural follow‐up, although some centers routinely dilate in a successive fashion (30–35 mm).

Esophageal functional luminal imaging probe (FLIP) represents a novel approach that combines volumetric dilation with functional assessment. Using an electro‐hydraulic computer‐controlled pump, EsoFLIP enables individualized balloon dilation across a wide range (12–30 mm) with precise control of fill volume and real time visual balloon shape. Esophagogastric junction (EGJ) distensibility index (DI), luminal diameter (Dia), cross‐sectional area (CSA), and balloon pressure (BP) can be measured directly before and after dilation via EndoFLIP, allowing immediate quantification of treatment effect on esophageal metrics [4, 5, 6, 7, 8, 9]. Its feasibility and short‐term clinical efficacy have been demonstrated across various esophageal motility disorders, including achalasia [10, 11, 12, 13].

FLIP metrics have been suggested to predict clinical outcomes after POEM, LHM, and PD with various results. Changes in EGJ‐DI of 1.8–3.8 mm^2^/mmHg [14, 15], percent changes of 272%–360% in DI and CSA [16], and absolute postinterventional values of 4.5–10 mm^2^/mmHg [4, 5] have been associated with symptomatic improvement, though optimal cut‐off values remain unclear and vary widely across studies. However, whether FLIP metrics can predict clinical outcomes after EsoFLIP dilation, and which parameters (DI, Dia, CSA, BP) perform best, remains unknown.

The aim of our study was to determine, in patients with achalasia undergoing EsoFLIP dilation, the impedance planimetry measurements that best predict short‐term clinical response.

## 2 Materials & Methods

### 2.1 Patients

This study was approved by the Zurich Ethical Committee (BASEC‐No. 2017‐00930). All patients provided written informed consent. We studied data of 138 consecutive patients with achalasia undergoing EsoFLIP dilation at the university hospital of Zurich Switzerland between January 2017 to October 2025. All high‐resolution manometry (HRM) was performed at the authors' institution. Studies were conducted by different examiners over the study period, but all tracings were interpreted according to the Chicago Classification (v3.0/v4.0) using standardized institutional criteria, ensuring consistent classification of achalasia subtypes. Testing regime at our center by default includes single water swallows, multiple rapid swallows, and a standardized rice test meal. Patients were excluded from the study if they had previous therapy of achalasia or previous hiatal or gastric surgery.

### 2.2 FLIP Protocol

EGJ dilation was performed using the EsoFLIP330 esophageal functional luminal imaging probe (EsoFLIP, Crospon Ltd./Medtronic), a hydraulic balloon dilation device that measures diameter and CSA in real time via impedance planimetry using 15 electrodes distributed along a 70‐mm segment of the catheter shaft (maximum balloon diameter 30 mm, ffES‐330). Saline is injected into the balloon via a computer‐controlled electrohydraulic pump. As the physical properties of the EsoFLIP catheter do not permit distensibility measurements and intraballoon pressure is integrated into the device, our standard approach includes the use of a diagnostic EndoFLIP catheter immediately before and approximately 3–5 min after EsoFLIP deflation (EndoFLIP 322‐16 cm panometry catheter, Medtronic) under standardized conditions (left‐lateral position, propofol sedation). In patients with achalasia or suspected outlet obstruction, the diagnostic EndoFLIP catheter is positioned across the EGJ under endoscopic guidance, after which the endoscope is withdrawn for FLIP measurements [17]. All measurements are obtained during balloon filling rather than deflation to minimize the potential influence of EGJ recoil on recorded values. The following standardized FLIP measurements are obtained: EGJ distensibility index (DI, calculated as CSA divided by intraballoon pressure, mm^2^/mmHg) and balloon pressure (BP, mmHg) at 60 mL, as well as EGJ diameter (Dia, mm) and CSA (mm^2^) at 70 mL, following sequential saline infusion to 50, 60, and 70 mL, with at least 30 s stabilization at each fill volume. All measurements are recorded in real time during the procedure rather than derived retrospectively from stored tracings. This protocol is consistent with the Dallas Consensus recommendations and with the joint American Gastroenterological Association/European Society of Neurogastroenterology and Motility practice guideline on the clinical use of FLIP [18, 19]. These two consensus frameworks are largely concordant with respect to catheter calibration, balloon positioning, fill‐volume protocol, pressure measurement, and stabilization time.

### 2.3 Study Outcomes

The primary outcome was symptomatic response at 4 weeks, assessed using the Eckardt score (ES), which ranges from 0 to 12 based on dysphagia, regurgitation, chest pain, and weight loss [20]. Both the conventional threshold (ES ≤ 3 or ≥ 3‐point reduction from baseline) and a more stringent threshold for excellent response (ES ≤ 1) were evaluated. Predictive modeling focused on ES ≤ 1 due to the limited discriminative performance of the conventional threshold. Objective response was assessed using timed barium esophagogram (TBE), defined as barium column height < 5 cm at 5 min. For each FLIP parameter (DI, BP, Dia, CSA), pre‐interventional values, postinterventional values, and delta values (Δ = postinterventional—pre‐interventional) were evaluated as potential predictors of symptomatic response.

### 2.4 Statistical Analysis

Continuous variables were summarized using mean ± standard deviation, and median with interquartile range (IQR), as appropriate. Pre‐ and post‐interventional differences were assessed using the paired Wilcoxon signed‐rank test. A *p*‐value 0.05 or less was considered statistically significant. Receiver operating characteristic (ROC) analyses were performed to assess the discriminative ability of baseline, post‐intervention, and within‐subject changes (delta) of each parameter for predicting the respective outcome, with AUROC calculated as a measure of overall discrimination. Optimal cut‐off values were determined under the constraint of a minimum sensitivity of 0.80, reflecting a clinically driven preference to prioritize detection of responders. Among thresholds with sensitivity ≥ 0.80, the cut‐off maximizing the Youden index was selected. Thresholds resulting in degenerate classifications (i.e., specificity of 0 or 1, corresponding to classifying all observations into a single category) were excluded to avoid trivial solutions. Robustness of model performance and cut‐off selection was assessed using stratified bootstrap resampling (*n* = 1000 iterations, preserving the original outcome class distribution), with AUROC, sensitivity, specificity, and balanced accuracy summarized as mean values and cut‐offs summarized by median, IQR, and 95% CI across resamples. Analyses were restricted to complete cases without missing values in predictors or outcomes.

## 3 Results

A total of 79 treatment‐naïve patients were included in the overall analysis, of whom 51 had complete pre‐ and post‐dilation FLIP data that were included in the predictive analysis (Figure 1). Baseline characteristics and clinical response for both cohorts are summarized in Table 1. For the conventional threshold (ES ≤ 3 or ≥ 3‐point reduction), FLIP metrics demonstrated insufficient discriminative performance in this sample size. Therefore, predictive analyses focused on the stringent threshold (ES ≤ 1). One patient (1.3%) with achalasia Type 2 developed a delayed esophageal perforation asymptomatic at discharge, vomited at home after eating, admitted with chest pain, identification of distal esophageal perforation (Boerhave‐type scenario).

**FIGURE 1:**
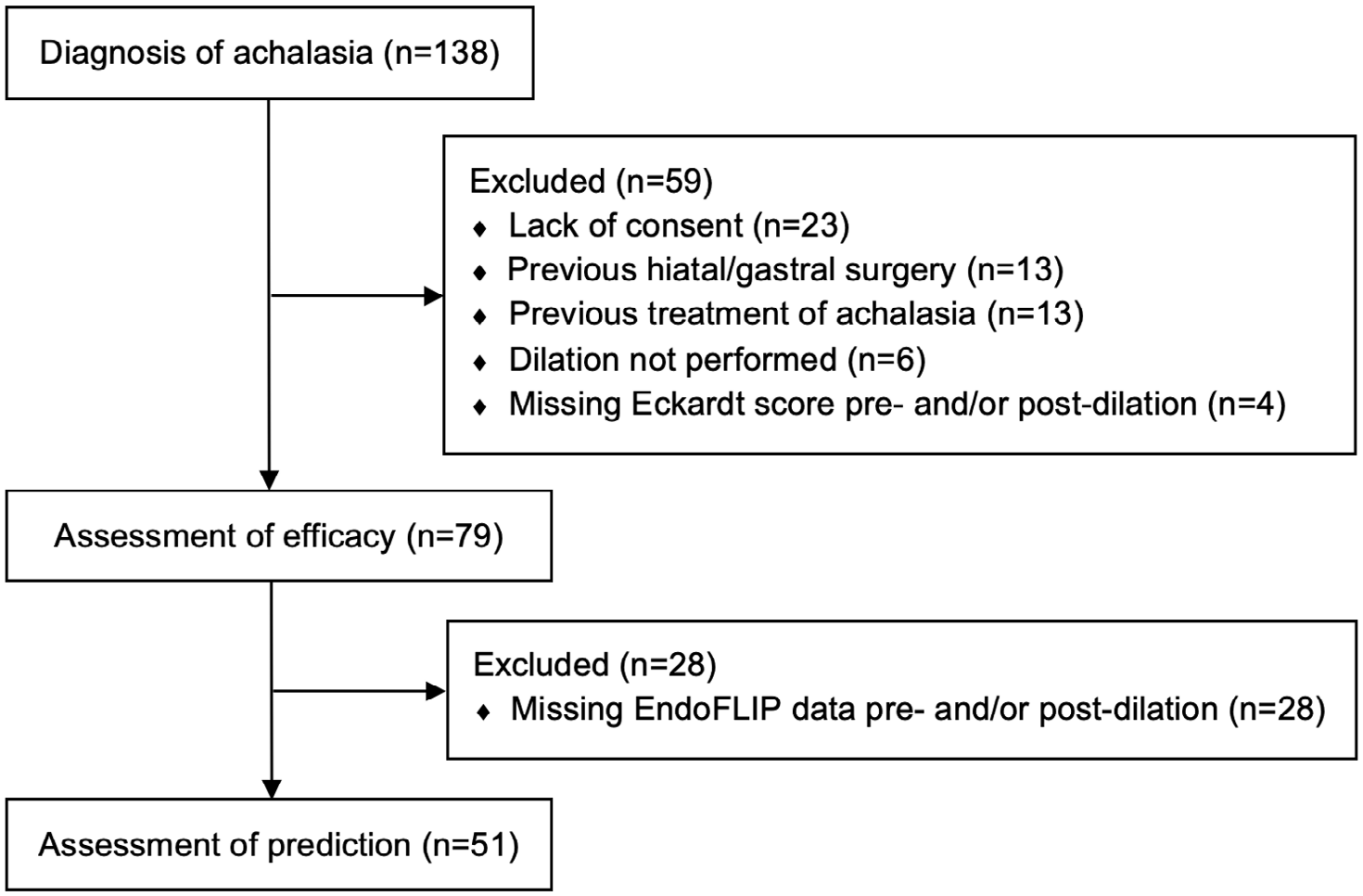
Flow diagram of study participants.

**TABLE 1:** Baseline characteristics and clinical response.

Characteristic	Overall cohort (*n* = 79)	Predictive subset (*n* = 51)
Female sex	38% (30/79)	43% (22/51)
Type I achalasia	8% (6/79)	10% (5/51)
Type II achalasia	87% (69/79)	84% (43/51)
Type III achalasia	5% (4/79)	6% (3/51)
Median age, years	52 (range 14–86)	57 (IQR 39–71)
Pre‐dilation ES, points	6 (IQR 4–7)	5 (IQR 4–6)
Post‐dilation ES, points	2 (IQR 0–4)	2 (IQR 0–3)
Pre‐dilation ES ≤ 3	18% (14/79)	22% (11/51)
Pre‐dilation ES ≤ 1	3% (2/79)	4% (2/51)
Conventional response (ES ≤ 3 or ΔES ≥ 3)	81% (64/79)	84% (43/51)
Stringent response (ES ≤ 1)	46% (36/79)	49% (25/51)
Objective response (TBE < 5 cm at 5 min)	67% (47/70)	66% (29/44)
Pre‐dilation EGJ diameter, mm	10 (IQR 8–11)	10 (IQR 8–11)
Post‐dilation EGJ diameter, mm	17 (IQR 15–18)	17 (IQR 15–18)

Abbreviations: EGJ, esophagogastric junction; ES, Eckardt score; IQR, interquartile range; TBE, timed barium esophagogram.

Median EGJ‐Dia70 increased from 10 mm (IQR 8–11) to 17 mm (IQR 15–18), EGJ‐CSA70 from 72 mm^2^ (IQR 49–102) to 220 mm^2^ (IQR 181–257), and EGJ‐DI from 1.4 mm^2^/mmHg (IQR 0.8–1.9) to 5.8 mm^2^/mmHg (IQR 4.6–7.6) all *p* < 0.001. Median EGJ‐BP60 decreased from 31 mmHg (IQR 24–38) to 24 mmHg (IQR 19–29, *p* < 0.001).

Within‐subject changes (delta values) in EGJ‐Dia70 and EGJ‐CSA70 demonstrated the highest predictive performance for short‐term clinical response, with AUROCs of 0.77 (95% CI 0.63–0.91) and 0.75 (95% CI 0.60–0.89), respectively (Figure 2). Cut‐offs of ΔEGJ‐Dia ≥ 6.3 mm and ΔEGJ‐CSA ≥ 129.3 mm^2^ predicted response with sensitivities/specificities of 0.80/0.47 and 0.80/0.61, respectively. ΔEGJ‐DI60 showed moderate predictive performance (AUROC 0.65, 95% CI 0.49–0.81; cut‐off ≥ 3.5 mm^2^/mmHg; sensitivity 0.81, specificity 0.44). In contrast, ΔBP60 demonstrated no meaningful discriminative ability (AUROC 0.45, 95% CI 0.29–0.62).

**FIGURE 2:**
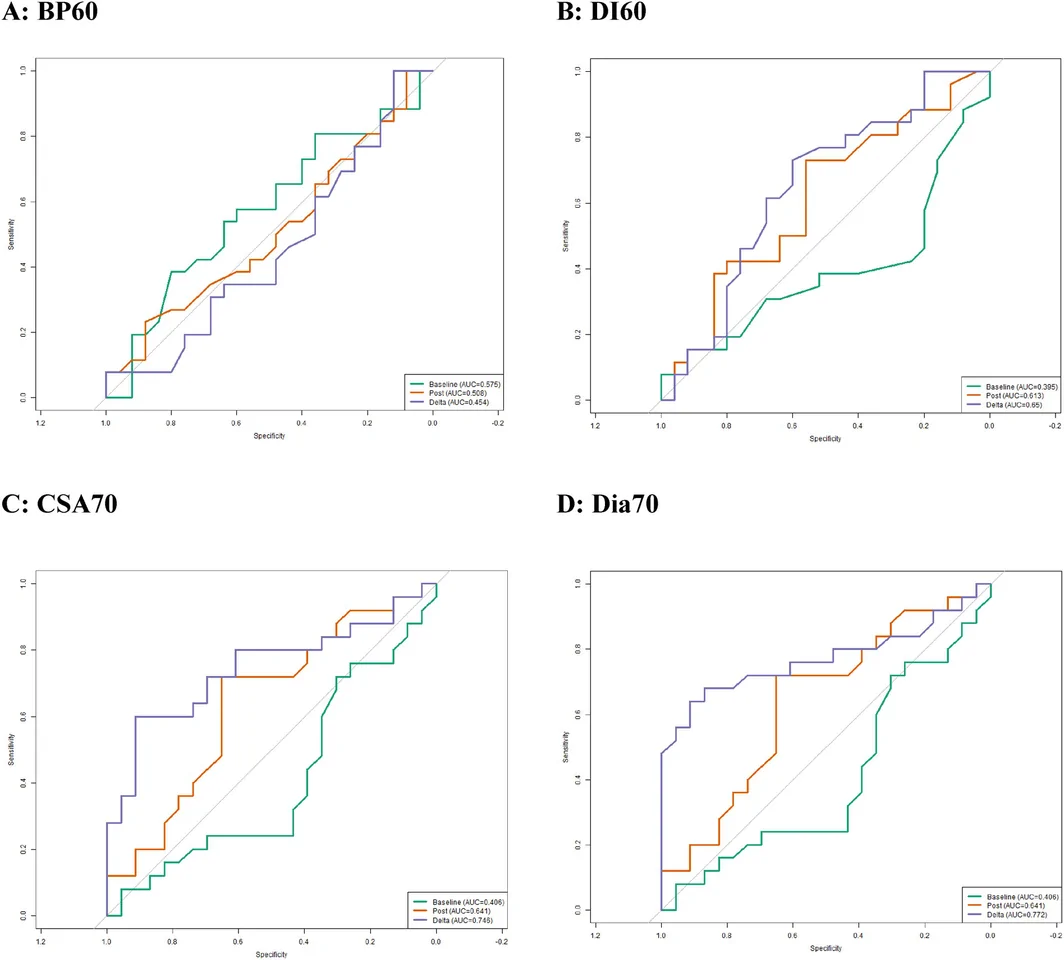
ROC analysis of FLIP parameter for predicting short‐term symptomatic response. (A): EGJ‐BP60, (B): EGJ‐DI60, (C): EGJ‐CSA70, (D): EGJ‐Dia70; each panel shows ROC curves for pre‐interventional (green), post‐interventional (orange), and delta values (violet).

Bootstrap resampling (*n* = 1000) supported the stability of these findings. For ΔEGJ‐Dia70, the bootstrap mean AUC was 0.77 (95% CI 0.62–0.90), with a consistent cut‐off across samples (median 6.3 mm; IQR 4.2–6.7) and sensitivity/specificity of 0.85/0.48. For ΔEGJ‐CSA70, the bootstrap mean AUC was 0.74 (95% CI 0.60–0.87), with moderate cut‐off variability (median 129 mm^2^; IQR 97–131) and sensitivity/specificity of 0.80/0.61.

Detailed predictive performance metrics for all FLIP parameters are summarized in the Supplementary Tables 1 and 2.

## 4 Discussion

In this study evaluating FLIP metrics after EsoFLIP dilation in treatment‐naïve patients with achalasia, within‐subject changes (delta values) demonstrated meaningful discriminative ability for predicting symptomatic response (ES ≤ 1). ΔEGJ‐Dia70 yielded the highest predictive accuracy with a cut‐off of 6.3 mm (AUC 0.77, 95% CI 0.63–0.91), followed by ΔEGJ‐CSA70 at 129.3 mm^2^ (AUC 0.75, 95% CI 0.60–0.89), and ΔEGJ‐DI60 at 3.5 mm^2^/mmHg (AUC 0.65, 95% CI 0.49–0.81). This study provides the first predictive FLIP data specifically after EsoFLIP dilation.

The symptomatic response rate was 81% using the conventional threshold (ES ≤ 3 or ≥ 3‐point reduction) after singular dilation, comparable to the 70%–90% reported for PD and POEM [11, 21]. The lower rate of 46% for ES ≤ 1 reflects the stringent definition chosen to identify excellent responders and provide optimal discriminative ability for predictive modeling. An objective response rate of 67% (TBE < 5 cm at 5 min) further supports clinical efficacy. These predictive FLIP metrics were derived using the stringent threshold (ES ≤ 1), whereas their discriminative performance was limited when the conventional threshold (ES ≤ 3 or ≥ 3‐point reduction) was applied. Because ES ≤ 1 and ES ≤ 3 classify different patients as responders and non‐responders, this difference in cohort composition likely explains the divergent discrimination, and the reported FLIP cut‐offs should be considered specific to the stringent response definition used here.

As shown in Supplementary Table 2, baseline FLIP metrics showed no meaningful discriminative ability for predicting treatment response (AUCs for BP60, DI60, CSA70, and Dia70: 0.57, 0.39, 0.41, and 0.41, respectively). However, baseline measurements remain useful for diagnosing achalasia. The Dallas Consensus [18] defines impaired EGJ opening by EGJ‐Dia70 < 12 mm and EGJ‐DI60 < 2.0 mm^2^/mmHg, criteria consistent with our median baseline values (EGJ‐Dia70 10 mm, EGJ‐DI60 1 mm^2^/mmHg).

Absolute post‐dilation FLIP values (Supplementary Table 2) demonstrated only moderate discriminative ability (post‐EGJ‐DI60 AUC 0.61, 95% CI 0.45–0.77, cut‐off 4.7 mm^2^/mmHg), comparable to the AUC of 0.56 reported by Wu et al. [14] after PD. Low post‐dilation EGJ‐DI (< 2.8–3.1 mm^2^/mmHg) has been associated with persistent dysphagia [6, 22], while higher values (4.5–8.5 mm^2^/mmHg) have been associated with reduced dysphagia at > 6 months after POEM or LHM [4]. Yoo et al. [5] suggested an optimal range of 7–10 mm^2^/mmHg after POEM. Conversely, excessively high values (EGJ‐DI ≥ 9.3 mm^2^/mmHg, EGJ‐CSA ≥ 96 mm^2^) have been associated with reflux esophagitis mmHg [23, 24], highlighting the need to balance adequate dilation against iatrogenic reflux risk. However, these studies used different fill volumes (30–40 mL vs. our 60–70 mL) and balloon systems (8 cm vs. 16 cm EndoFLIP), limiting direct comparability.

Notably, Jain et al. [6] reported that low absolute post‐interventional EGJ‐DI values were highly predictive (AUC 0.90) of objective treatment failure (TBE barium retention), but only in patients with normal esophageal anatomy. In patients with anatomic deformities (pseudodiverticula, sigmoid esophagus, epiphrenic diverticula, or sinktrap; 34% of the cohort), FLIP metrics did not predict retention, suggesting that abnormal anatomy modulates treatment success through mechanisms independently of EGJ distensibility.

Our results demonstrate that within‐subject changes (delta values) are predictive of short‐term outcomes, consistent with previous findings after PD, POEM, and LHM [6, 14, 15]. Our ΔEGJ‐Dia cut‐off of 6.3 mm is lower than the 8.2 mm threshold reported by Kamboj et al. [15], and falls between the responder (8.6 mm) and non‐responder (4.6 mm) values reported by Jain et al. [6]. Similarly, our ΔEGJ‐DI cut‐off of 3.5 mm^2^/mmHg is comparable to the 3.8 mm^2^/mmHg threshold reported by Kamboj et al. [15], though higher than the 1.8 mm^2^/mmHg cut‐off reported by Wu et al. [14] after PD (AUC 0.89). The lower AUC for ΔEGJ‐DI in our study (0.65 vs. 0.89) may reflect differences in fill volumes (60 mL vs. 40 mL), outcome definition (ES ≤ 1 vs. ≤ 3), and intervention type (EsoFLIP vs. PD).

Our data suggest that ΔEGJ‐CSA70 and ΔEGJ‐Dia70 may serve as an additional useful parameter for predicting symptomatic response. Ngamruengphong et al. [8] demonstrated that intraoperative EGJ‐CSA correlated with clinical response after POEM (89.0 vs. 72.4 mm^2^ for good vs. poor response, *p* = 0.01). Moran et al. [16] found that percent change in CSA was the most predictive metric for clinical response after POEM (AUC 0.75), with an optimal cut‐off of 360% change. Our finding that ΔEGJ‐CSA70 predicts symptomatic response (cut‐off 129.3 mm^2^, AUC 0.75) adds to this emerging evidence that CSA may provide complementary prognostic information beyond EGJ‐DI alone, particularly at higher fill volumes where pressure may plateau. In contrast, BP is primarily used as the denominator in the DI calculation (DI = CSA/BP) rather than as an independent predictive metric, and the literature does not establish BP cut‐offs for predicting treatment outcomes. Accordingly, our results showed poor predictive value for ΔBP60 (AUC 0.45).

### 4.1 Clinical Implications

A ΔEGJ‐Dia < 6.3 mm or ΔEGJ‐CSA < 129.3 mm^2^ following EsoFLIP dilation was associated with less favorable short‐term symptomatic response and may help identify patients unlikely to benefit from a single dilation session. Given the moderate specificity of these thresholds, these findings are regarded as hypothesis‐generating and should be tested by other groups to validate this as a basis for intraprocedural decision‐making (e.g., re‐dilation to a larger diameter during the same session). Pre‐ and post‐dilation panometry measures can similarly be obtained by bracketing conventional PD with an EndoFLIP study. The potential advantage of EsoFLIP over PD lies in its stepwise, continuously variable hydraulic dilation and real‐time visualization of balloon geometry and EGJ morphology, allowing individualized control of the dilation process. In addition, the fluid‐filled EsoFLIP balloon provides improved endoscopic visualization during dilation, and its less bulky, more flexible profile facilitates smoother passage through the larynx and EGJ without the need for fluoroscopic guidance or a guidewire. From a cost perspective, the EsoFLIP balloon is considerably less expensive than a standard Rigiflex balloon, which may further support its adoption in clinical practice.

The reported sample exhibited an observed perforation rate (1/79, 1.3%) at the lower end of published estimates for pneumatic dilation at experienced centers (1.9%) [2]. A larger cohort would be necessary to display safety.

Whether this controlled, graded approach provides clinically meaningful advantages over established therapies (e.g., PD, POEM) remains to be determined. Since these thresholds were derived from short‐term outcomes only (4 weeks), their relationship to longer‐term durability is unknown. Prospective studies with longer follow‐up are needed before these cut‐offs can be applied to inform a “tailored therapy” approach.

### 4.2 Strengths and Limitations

This study has several strengths. First, it is the first to systematically evaluate predictive FLIP metrics after EsoFLIP dilation, addressing a gap in the literature where previous studies focused on POEM, LHM, and PD. Second, adherence to Dallas Consensus protocol (50–60–70 mL) [18] ensures standardized measurements and improved comparability with future studies. Third, bootstrap validation confirmed the robustness of the predictive models. Fourth, given the relative rarity of achalasia, the sample size of 79 treatment‐naïve patients represents a substantial cohort, comparable to or larger than existing studies in this emerging field.

Several limitations should be acknowledged. First, this study was conducted at a single center by a single experienced endoscopist, with a predominantly Type II achalasia cohort (87%), which may limit generalizability to other achalasia subtypes, operators, and centers. However, both the EndoFLIP and EsoFLIP protocols were highly standardized in this study, which should facilitate comparability with future studies. External validation of the proposed thresholds in multicenter cohorts with broader subtype representation is needed before clinical application. Second, direct comparison with earlier studies is limited due to differences in balloon systems (16 cm vs. 8 cm EndoFLIP catheters), fill volumes (60–70 mL vs. 30–40 mL), and outcome definitions (ES ≤ 1 vs. ES ≤ 3). Third, while TBE data were collected, the analysis focused exclusively on symptomatic response (ES ≤ 1) due to incomplete TBE datasets. Fourth, follow‐up duration of 4 weeks is likely insufficient to assess long‐term treatment durability; longer‐term outcomes will be evaluated in a future analysis. Fifth, standardized pre‐ and post‐dilation EndoFLIP measurement was implemented only from 2020 onward, accounting for the incomplete FLIP datasets. Consequently, only 65% (51/79) of patients had complete pre‐ and post‐dilation FLIP data available for the predictive analysis. However, baseline characteristics and clinical response were comparable between the overall cohort and this predictive subset (Table 1), arguing against substantial selection bias.

## 5 Conclusion

This study provides the first systematic evaluation of predictive FLIP metrics after EsoFLIP dilation in treatment‐naïve patients with achalasia. Delta values, particularly ΔEGJ‐Dia70 (cut‐off 6.3 mm, AUC 0.77) and ΔEGJ‐CSA70 (cut‐off 129.3 mm^2^, AUC 0.75), were predictive of short‐term symptomatic response (ES ≤ 1), while absolute postinterventional values showed limited discriminative ability. These cut‐offs may serve as procedural targets to guide adequate dilation. Future studies should validate these thresholds in larger cohorts and assess long‐term outcomes.

## Author Contributions

J.S.: Drafting of manuscript, analysis and interpretation of data, acquisition of data, study concept and design; V.S.: Analysis and interpretation of data, critical revision of manuscript, study concept and design; J.Sc.: Acquisition of data, critical revision of manuscript; J.M.: Acquisition of data, critical revision of manuscript; A.B.: Acquisition of data, critical revision of manuscript; D.P.: Analysis and interpretation of data, critical revision of manuscript, study concept and design, FLIP examination.

## Funding

The authors have nothing to report.

## Disclosure

Daniel Pohl is a consultant/speaker to Medtronic, Schwabe Pharma and board member of DigeHealth.

## Conflicts of Interest

The authors declare no conflicts of interest.

## Supporting information


**Supplementary Table 1.** Predictive values of within‐subject changes (delta) of impedance planimetry measurements.
**Supplementary Table 2**. Predictive values of pre‐dilation and post‐dilation impedance planimetry measurements.

## Data Availability

The data that support the findings of this study are available from the corresponding author upon reasonable request.
