# Supplementary material for: FLIP Measurements Predict Short‐Term Outcomes After Singular EsoFLIP Dilation in Patients With Achalasia
Source: Neurogastroenterol Motil. 2026 Nov 9;38(11):e70500. doi: 10.1111/nmo.70500 (PMC13749055; doi:10.1111/nmo.70500)
Supplement: Supplementary file 1 [file NMO-38-e70500-s001.docx]

**Appendix**

**Supplementary Table 1. Predictive values of within-subject changes (delta) of impedance planimetry measurements**

| FLIP measurement | AUC (95% CI) | Sensitivity / Specificity | PPV / NPV | Cut-off |
| --- | --- | --- | --- | --- |
| ΔBP60 | 0.45 (0.29-0.62) | 1.00 / 0.12 | 0.54 / 1.00 | -27.5 mmHg |
| ΔDI60 | 0.65 (0.49-0.81) | 0.81 / 0.44 | 0.60 / 0.69 | 3.5 mm^2^/mmHg |
| ΔCSA70 | 0.75 (0.60-0.89) | 0.80 / 0.61 | 0.69 / 0.74 | 129.3 mm² |
| ΔDia70 | 0.77 (0.63-0.91) | 0.80 / 0.48 | 0.63 / 0.69 | 6.3 mm |

AUC, area under the curve; BP, balloon pressure; DIA, diameter; DI, distensibility index; CI, confidence interval; CSA, cross-sectional area; NPV, negative predictive value; PPV, positive predictive value

**Supplementary Table 2. Predictive values of pre-dilation and post-dilation impedance planimetry measurements**

| FLIP measurement | AUC (95% CI) | Sensitivity / Specificity | PPV / NPV | Cut-off |
| --- | --- | --- | --- | --- |
| PreBP60 | 0.57 (0.41-0.74) | 0.81 / 0.36 | 0.57 / 0.64 | -25.1 mmHg |
| PreDI60 | 0.39 (0.23-0.56) | 0.89 / 0.08 | 0.50 / 0.40 | 0.6 mm^2^/mmHg |
| PreCSA70 | 0.41 (0.24-0.57) | 0.88 / 0.09 | 0.50 / 0.40 | 34.7 mm² |
| PreDia70 | 0.41 (0.24-0.57) | 0.88 / 0.09 | 0.50 / 0.40 | 6.7 mm |
|  | | | | |
| PostBP60 | 0.51 (0.35-0.67) | 1.00 / 0.08 | 0.53 / 1.00 | -13.2 mmHg |
| PostDI60 | 0.61 (0.45-0.77) | 0.81 / 0.36 | 0.57 / 0.64 | 4.7 mm^2^/mmHg |
| PostCSA70 | 0.64 (0.48-0.80) | 0.80 / 0.39 | 0.59 / 0.64 | 182.7 mm² |
| PostDia70 | 0.64 (0.48-0.80) | 0.80 / 0.39 | 0.58 / 0.64 | 15.3 mm |

AUC, area under the curve; BP, balloon pressure; DIA, diameter; DI, distensibility index; CI, confidence interval; CSA, cross-sectional area; NPV, negative predictive value; PPV, positive predictive value
